# Whole-body MRI for staging and follow-up of primary musculoskeletal tumours: a systematic review

**DOI:** 10.1093/bjr/tqag009

**Published:** 2026-01-08

**Authors:** Domenico Albano, Sergio Garziano, Moreno Zanardo, Gaia Ghilardi, Carlotta Casale, Salvatore Gitto, Carmelo Messina, Angelo Vanzulli, Luca Maria Sconfienza

**Affiliations:** Department of Radiology, ASST Grande Ospedale Metropolitano Niguarda, Milan 20162, Italy; Dipartimento di Scienze Biomediche, Chirurgiche ed Odontoiatriche, Università Degli Studi di Milano, Milan 20122, Italy; Scuola di Specializzazione in Radiodiagnostica, Università degli Studi di Milano, Milan 20122, Italy; Radiology Unit, IRCCS Policlinico San Donato, San Donato Milanese 20097, Italy; Scuola di Specializzazione in Radiodiagnostica, Università degli Studi di Milano, Milan 20122, Italy; Scuola di Specializzazione in Radiodiagnostica, Università degli Studi di Milano, Milan 20122, Italy; Unit of Diagnostic and Interventional radiology, IRCCS Istituto Ortopedico Galeazzi, Milan 20157, Italy; Dipartimento di Scienze Biomediche per la Salute, Università degli Studi di Milano, Milan 20133, Italy; Dipartimento di Scienze Biomediche per la Salute, Università degli Studi di Milano, Milan 20133, Italy; UOC Radiodiagnostica, ASST Centro, Specialistico Ortopedico Traumatologico Gaetano Pini-CTO, Milan 20122, Italy; Department of Radiology, ASST Grande Ospedale Metropolitano Niguarda, Milan 20162, Italy; Department of Oncology and Hemato-Oncology, Università degli Studi di Milano, Milan 20122, Italy; Unit of Diagnostic and Interventional radiology, IRCCS Istituto Ortopedico Galeazzi, Milan 20157, Italy; Dipartimento di Scienze Biomediche per la Salute, Università degli Studi di Milano, Milan 20133, Italy

**Keywords:** whole body staging, magnetic resonance imaging, bone tumour, soft tissue tumour, sarcoma, systematic review

## Abstract

**Objectives:**

To evaluate the performance and technical parameters of whole body (WB)-MRI for staging and follow-up of primary musculoskeletal tumours.

**Methods:**

A systematic review was done in PubMed and Embase through July 2025. Eligible studies reported WB-MRI for staging or follow-up of bone/soft tissue sarcomas. Extracted data were study design, patient characteristics, MRI protocols, scan duration, and diagnostic performance. Methodological quality was assessed with QualSyst.

**Results:**

A total of 10 studies, published between 2016 and 2024, were included from 432 records. Most were retrospective (90%), with study populations ranging from 9 to 319 patients (total *n = *790, age range 2-80 years). Half of the studies focused on myxoid liposarcoma, while others addressed osteosarcoma, Ewing sarcoma, and chondrosarcoma. WB-MRI protocols employed 1.5T and/or 3T scanners. Non-contrast protocols (8/10 studies) mostly included T1 and STIR sequences. Exam durations ranged from 30 to 78 minutes, with outliers up to 250 minutes. Reference standards included CT, PET-CT, and bone scintigraphy. Diagnostic accuracy was investigated in only 2 studies, reporting 100% sensitivity, 96.3% specificity, and 97.3% accuracy for extrapulmonary metastases, 83%-88% sensitivity and 94%-95% specificity for bone metastases. The studies demonstrated high methodological rigour, with scores ranging from 16 to 19 out of 20.

**Conclusions:**

WB-MRI is a feasible and promising modality for staging and follow-up of primary musculoskeletal tumours. Evidence is still limited, based on heterogeneous and mostly retrospective studies. Larger, prospective, and standardized studies are needed to validate its accuracy, optimize imaging protocols and clarify its role in sarcoma imaging.

**Advances in knowledge:**

WB-MRI has been tested scarcely as a comprehensive, non-ionizing alternative for whole-body staging in selected patients with primary musculoskeletal tumours, mostly using unenhanced T1 and STIR sequences, with limited use of contrast media. Current evidence is insufficient to recommend routine use of WB-MRI, standardized protocols and prospective validation are needed.

## Introduction

Imaging for staging and follow-up of malignant tumours represents a critical component of patient management, enabling accurate determination of the initial disease burden, guiding therapeutic decision-making, evaluating treatment response, and providing prognostic insights. A variety of whole-body imaging modalities are routinely employed in both the diagnostic work-up and longitudinal monitoring of oncologic patients. These include established techniques such as computed tomography (CT), positron emission tomography-CT (PET-CT), bone scintigraphy, and other nuclear medicine procedures, as well as non-ionizing approaches such as whole-body magnetic resonance imaging (WB-MRI).^1–3^

WB-MRI has gained increasing clinical relevance in the evaluation of several malignancies, most notably prostate cancer, lymphoma, and multiple myeloma, but also melanoma, breast, ovarian, colorectal, and lung carcinomas.^1^^,^^4–7^ Furthermore, it has been applied in the screening and surveillance of paediatric patients with cancer-predisposing syndromes.^8^^,^^9^ Its adoption is largely driven by the capacity to provide comprehensive, repeatable assessments while avoiding the risks associated with ionizing radiation exposure—an especially important consideration in younger patients.^1^

Despite MRI being firmly established as the reference standard for assessing the loco-regional extent of primary musculoskeletal tumours—often complementing X-ray and CT due to its superior soft tissue contrast and high sensitivity in detecting bone marrow involvement—its role in extra-regional staging remains limited. To date, relatively few studies have systematically evaluated the clinical utility and technical aspects of WB-MRI in this specific context.^1^^,^^10^^,^^11^

The present systematic review aims to critically examine the performance and technical parameters of WB-MRI in the initial staging and follow-up of primary musculoskeletal tumours.

We will focus on the characteristics of different pulse sequences, the role of contrast administration, and strategies for optimizing examination duration, with the goal of providing a comprehensive overview to support clinical practice.

## Methods

### Study design

This systematic review was conducted in accordance with the Preferred Reporting Items for Systematic Reviews and Meta-Analyses (PRISMA).^12^ The protocol for this systematic review has been registered in the International Prospective Register of Systematic Reviews (PROSPERO) database (registration unique identifying number: CRD420251139293).

### Literature search strategy

A systematic search was conducted in PubMed/MEDLINE and Embase from inception to July 2025. The search combined controlled vocabulary (MeSH/Emtree) and free-text terms related to whole-body MRI and musculoskeletal tumours. No language or publication date restrictions were applied. The initial screening of studies was conducted by 1 reviewer (S.G.), a 4th year radiology resident with 2 years of experience in conducting systematic reviews, based on titles and abstracts, followed by a full-text review of potentially eligible articles. All titles and abstracts were independently screened by 2 reviewers (S.G. and G.G.), and full texts of potentially eligible studies were assessed in duplicate. In cases of disagreement, the original article files were re-examined in detail to verify the information and reach a shared decision. When uncertainties persisted, the issue was discussed during a consensus meeting that also involved the senior author to ensure methodological consistency and final agreement. In addition, the reference lists of all included studies and relevant review articles were manually examined to identify further eligible publications. The search process was performed by a reviewer with 2 years of experience in conducting systematic reviews and was supervised and validated by a senior researcher with ten years of expertise in the field. The detailed search strings used for PubMed/MEDLINE and Embase are reported as Supplementary Material.

To ensure comprehensive coverage, we designed a structured search strategy combining controlled vocabulary (MeSH/Emtree terms) and free-text keywords for both population (primary musculoskeletal tumours, including bone and soft tissue sarcomas) and intervention (whole-body MRI). The strategy was adapted for each database according to its indexing system and syntax requirements.

### Inclusion and exclusion criteria

The following inclusion criteria were used to select the studies: (1) research involving human participants; (2) studies evaluating the use of whole-body MRI for initial staging or follow-up of primary musculoskeletal tumours (bone and soft-tissue), irrespective of whether diagnostic accuracy metrics were reported, provided that imaging protocols and reference standards were described. Exclusion criteria were as follows: (1) case reports, case series, narrative reviews, clinical guidelines, expert consensus statements, editorials, letters to the editor, comments, and conference abstracts; (2) studies lacking sufficient data, particularly those without information on the number of patients, acquisition protocol, magnetic field strength, and included histotypes.

### Data extraction and analysis

Data extraction was carried out by 2 independent reviewers (S.G. and G.G.) using a standardized data collection form in Microsoft Excel. The following information was extracted: first author, year of publication, study design (prospective or retrospective), total sample size, demographic characteristics (mean or median age), tumour type, MRI unit field strength, total MRI duration, use of contrast agent, MRI sequences, follow-up duration, reference standard, sensitivity, specificity, and accuracy.

A descriptive statistical analysis was conducted to summarize the characteristics of the studies included. Extracted data were reported in tables form. Frequencies and percentages were used to describe categorical variables, while ranges were reported for continuous variables.

### Quality assessment

The methodological quality of the included studies was independently assessed by 2 reviewers (C.C. and D.A.) using the QualSyst Tool for the Quality Assessment of Primary Research Studies.^13^ This tool comprises 10 items evaluating various aspects of study quality, with each item scored as 0 (not described), 1 (inadequately described), or 2 (adequately described). Discrepancies between reviewers were resolved through consensus discussion.

## Results

### Study selection and characteristics

A total of 414 records were identified through the search strategy and 18 from the reference lists of eligible articles. After title and abstract screening, 304 records were excluded.

**Figure 1.**
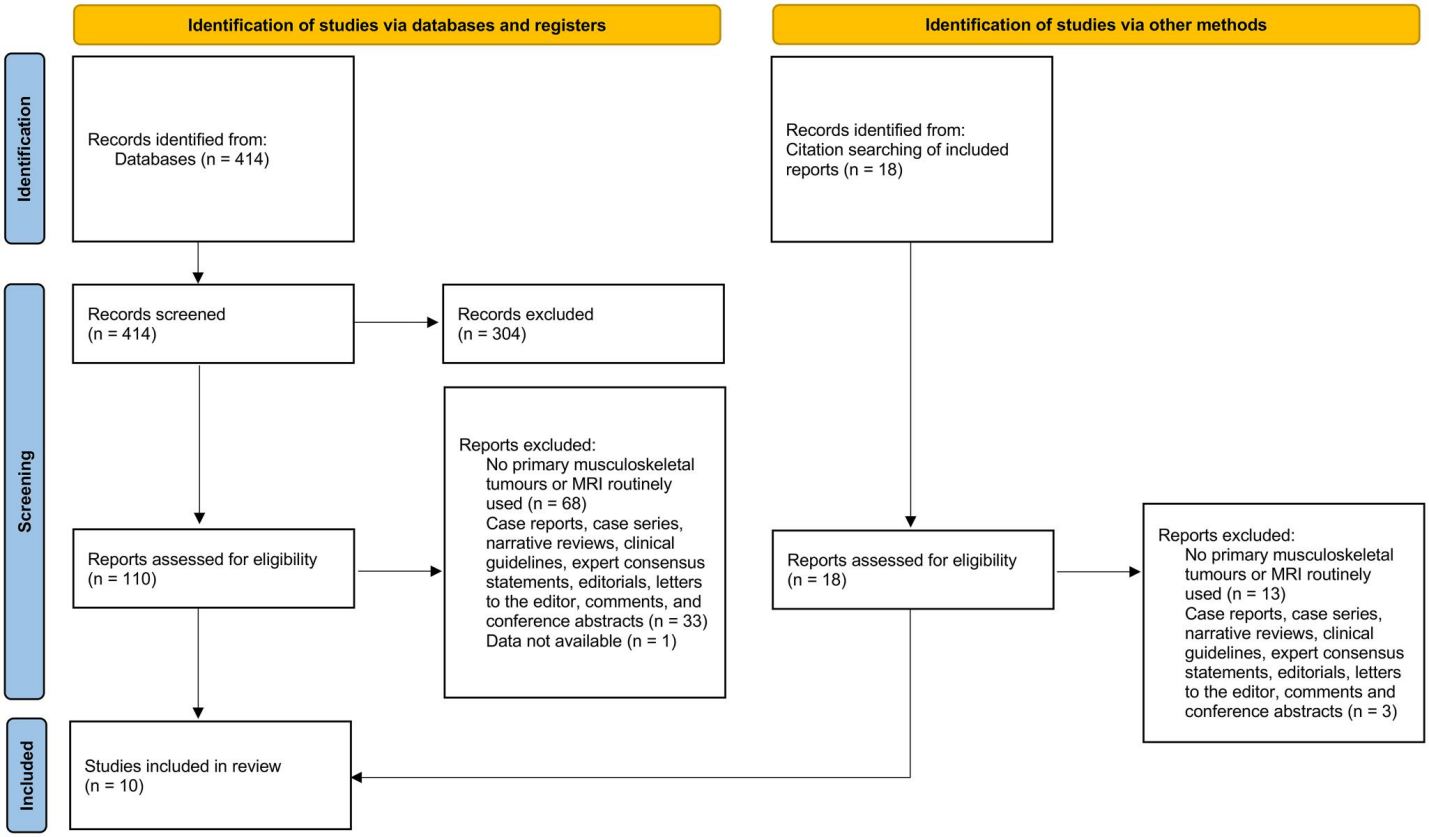
Flowchart depicting the study selection process, according to PRISMA.^12^

Of the 110 records assessed in full text, 8 met the inclusion criteria and were included in the systematic review. An additional 2 studies identified from reference lists were also included, resulting in a final total of 10. Figure 1 summarizes the study selection process.

The included articles were published between 2016^16^ and 2024.^14^ Nine studies (90%) had a retrospective design, while only 1 study (10%) was prospective.^15^ The corresponding authors were based in the UK^11^^,^^16^ and France^17^^,^^18^ in 2 studies each, while others were from the Netherlands,^14^ Denmark,^19^ Turkey,^3^ India,^15^ Canada,^10^ and the USA.^20^ One study was written in French and partially translated into English.^18^ Study populations ranged from 9^20^ to 319 patients,^14^ with a total of 790 patients and a median sample size of 40.5. Patients’ ages ranged from 2 years^11^ to 80 years.^18^ In 1 study, mean age was not reported.^20^ One study did not report participants’ mean age or gender distribution, and the supplementary material was not available. The gender distribution across the 10 included studies ranged widely. Most studies reported both female and male participants, with values ranging from F = 10/M = 12^18^ to F = 179/M = 140.^14^ Half of the studies (5/10) focused on myxoid liposarcoma.^10^^,^^16–18^^,^^20^ Two studies focused on osteosarcoma,^3^^,^^15^ Ewing sarcoma,^11^^,^^15^ and chondrosarcoma.^14^^,^^19^

### WB-MRI study protocol, acquisition parameters, and reference standard

All included studies used high-field MRI scanners: 5/10 (50%) employed 1.5T machines,^14–17^^,^^19^ 4/10 (40%) used both 1.5T and 3T scanners,^3^^,^^10^^,^^11^^,^^20^ and 1/10 used only 3T.^18^

A combination of MRI sequences was applied in all protocols:

1. In 8/10 studies without contrast administration, the most common sequences were T1 and STIR,^10^^,^^11^^,^^16^^,^^19^ followed by T1, T2 and STIR^3^^,^^14^^,^^17^ and STIR with diffusion-weighted imaging (DWI).^15^ Reported scan times ranged from 30 minutes^11^ to 78 minutes,^3^ with a median of 54 minutes. In the STIR/DWI protocol the exam duration time was not reported.
2. In 2/10 studies with contrast administration, more complex protocols were applied.

A study^18^ used STIR, T1 SE, T2 DIXON, and 3D T1 DIXON pre- and post-contrast sequences (Prohance 0.2 mL/kg) with a total exam duration time of 50 minutes. A study^20^ included T1, STIR, and FS T1 pre- and post-contrast sequences for initial staging with an exam time of 250 minutes, which was not well tolerated by patients and thus divided into 2-4 sessions. For follow-up, the same study used a STIR-only protocol (41 minutes). The name of the contrast agent was not reported.

Reference standards were not uniformly applied across studies; however, most included CT, PET-CT, and bone scintigraphy.^3^^,^^10^^,^^11^^,^^15^^,^^16^^,^^18^^,^^20^ Reported follow-up periods, when available, ranged widely from 6 months^3^ to 6 years.^20^

Diagnostic performance data were available in only 2 studies:

1. For extrapulmonary metastases from osteosarcoma, Karaarslan et al^3^ reported a sensitivity of 100% (95% CI: 69.15%-100%), a specificity of 96.3% (95% CI: 81.03%-99.91%), and an accuracy of 97.30% (95% CI: 85.84%-99.91%);
2. For bone metastases, Aryal et al^16^ reported a sensitivity of 83% (95% CI: 36%-100%) in osteosarcoma and 88% (95% CI: 47%-100%) in Ewing sarcoma, with a specificity of 94% (95% CI: 73%-100%) and 95% (95% CI: 77%-100%), respectively.

Table 1 summarizes the characteristics of the included studies.

**Table 1.** Characteristics of the included studies.

Study author	Country	Study design	Subjects	Gender	Age	Disease	MRI field and scan time	Contrast agent (Y/N)	Sequences	WB-MRI use
Van der Woude et al (2024)	Netherlands	Retrospective	319	F = 179, M = 140	16-79 y	Chondrosarcoma	1.5T, 45 min	N	T1, T2, STIR	Screening and follow-up
Jurik et al (2020)	Denmark	Retrospective	62	F = 36, M = 26	12-74 y	Chondrosarcoma	1.5T, 60 min	N	T1, STIR	Follow-up
Kalus and Saifuddin (2019)	UK	Retrospective	182	F = 56, M = 126	2-56 y	Ewing sarcoma	1.5/3T, 30 min	N	T1, STIR	Staging
Dewaguet et al (2023)	France	Retrospective	22	F = 10, M = 12	18-80 y	Myxoid liposarcoma	3T, 50 min	Y	T1, STIR, T2 DIXON, T1 DIXON pre and post-contrast	Staging
Stevenson et al (2016)	UK	Retrospective	28	F = 11, M = 17	23-59 y	Myxoid liposarcoma	1.5T, 60 min	N	T1, STIR	Staging and restaging
Karaarslan et al (2023)	Turkey	Retrospective	36	F = 13, M = 23	9-29 y	Osteosarcoma	1.5T (54-68 min); 3T (62-78 min)	N	T1, T2, STIR	Staging and restaging
Aryal et al (2021)	India	Prospective	54	F = 16, M = 38	Osteosarcoma: 17 (±7) y; Ewing sarcoma: 15 (±8) y	Osteosarcoma and Ewing sarcoma	1.5T; scan time N.A.	N	STIR, DWI	Staging
Gorelik et al (2018)	Canada	Retrospective	33	F = 13, M = 20	21-77 y	Myxoid liposarcoma	1.5T/3T 40-50 min	N	T1, STIR	Staging and follow-up
Chien et al (2019)	USA	Retrospective	9	NA	NA	Myxoid liposarcoma	1.5T/3T 250 min for staging (divided into 2-4 days); 41 min for follow-up	Y (staging)	T1, STIR, pre and post-contrast T1 for staging; STIR-only for follow-up	Staging and follow-up
Gouin et al (2019)	France	Retrospective	45	F = 12, M = 33	Non metastatic: 22-74 y; metastatic: 22-66 y	Myxoid liposarcoma	1.5T, 30-40 min	N	T1, STIR	Follow-up

### Quality assessment of included studies

The studies demonstrated high methodological rigour, with scores ranging from 16 to 19 out of 20. Studies by Kalus and Saifuddin,^11^ Aryal et al^16^ and Gouin et al^17^ obtained near-perfect scores, reflecting a high level of compliance with scientific standards. Nonetheless, a few studies—such as those by Jurik et al,^19^ Dewaguet et al,^18^ and Karaarslan et al^3^—received lower ratings because their reporting on data collection procedures and sampling approaches was less comprehensive. Table 2 reports full data of quality assessment.

**Table 2.** Quality assessment of the selected studies using the QualSyst Tool for the Quality Assessment of Primary Research Studies.^13^

Criteria	Question/objective sufficiently described?	Study design evident and appropriate?	Context for the study clear?	Connection to a theoretical framework/wider body of knowledge?	Sampling strategy described, relevant, and justified?	Data collection methods clearly described and systematic?	Data analysis clearly described and systematic?	Use of verification procedure(s) to establish credibility?	Conclusions supported by the results?	Reflexivity of the account?	Total
Van der Woude et al (2024)	2	2	2	2	1	2	1	1	2	1	**17**
Jurik et al (2020)	2	2	2	1	2	2	2	1	2	1	**16**
Kalus and Saifuddin (2019)	2	2	2	1	2	2	2	1	2	2	**19**
Dewaguet et al (2023)	2	2	2	2	2	2	2	1	2	1	**16**
Stevenson et al (2016)	2	2	2	1	2	2	2	1	2	1	**18**
Karaarslan et al (2023)	2	2	2	1	2	2	2	1	2	1	**16**
Aryal et al (2021)	2	2	2	2	2	2	2	2	2	2	**19**
Gorelik et al (2018)	2	2	2	2	2	2	2	1	2	2	**18**
Chien et al (2019)	2	2	2	2	1	1	2	2	2	2	**17**
Gouin et al (2019)	2	2	2	2	2	2	2	2	2	2	**19**

## Discussion

This systematic review evaluated the technical parameters and diagnostic performance of WB-MRI for the initial staging and follow-up of primary musculoskeletal tumours, with a particular emphasis on imaging protocols. According to our study, the available evidence concerning the application of WB-MRI for primary musculoskeletal malignancies remains limited compared to its well-established use in haematologic malignancies, prostate cancer, and paediatric oncology.^1^^,^^7^^,^^8^^,^^21^

Most included studies were retrospective, with only 1 prospective investigation, highlighting the generally low level of evidence in this field. Study populations were heterogeneous with respect to both tumour histology and patient age, ranging from paediatric to elderly cohorts. Myxoid liposarcoma was the most frequently investigated entity, reflecting the potential role of WB-MRI in whole-body staging of these tumours.^10^^,^^16–18^^,^^20^ Conversely, osteosarcoma, Ewing sarcoma, and chondrosarcoma were less commonly addressed, underscoring the need for more dedicated prospective studies across different tumour subtypes. All studies employed high-field MRI scanners, most commonly at 1.5T, with some including 3T acquisitions. This reflects current clinical landscape, where 1.5T scanners remain the most widely available. Furthermore, some radiologists may prefer to use 1.5T over 3T scanners to minimize potential artefacts encountered in whole-body imaging, particularly those related to respiration, air-tissue interfaces, and motion of the heart, vessels, and bowel.^22^

Most protocols largely relied on unenhanced sequences, especially T1-weighted and STIR imaging. The central role of STIR is unsurprising given its robust sensitivity for detecting marrow and soft tissue abnormalities, making it indispensable in WB-MRI protocols.^3^^,^^10^^,^^11^^,^^16^^,^^19^ The use of DWI, reported in only 1 study,^15^ may provide added value in oncologic applications by detecting marrow infiltration and distinguishing viable tumour tissue from post-treatment changes. However, its limited use in primary musculoskeletal tumours likely may reflect concerns about long acquisition times, susceptibility artefacts, and the need for further validation. Moreover, ADC values substantially overlap between benign and malignant bone tumours as well as among myxoid and fatty soft tissue tumours.^23^^,^^24^

Only 2 of the included studies employed contrast-enhanced protocols.^18^^,^^20^ In both, contrast administration prolonged examination times considerably, with 1 protocol reaching an impractical 250 minutes.^20^ Such long scan times clearly limit clinical feasibility, particularly in paediatric patients. Long acquisition times may lead to motion artefacts, reduced compliance, and limited clinical uptake. Although acceleration techniques offer promise for shortening scan duration, current evidence does not support the routine use of contrast in WB-MRI for musculoskeletal tumours. Given patient tolerance, costs and the small but potential risks associated with gadolinium administration, our findings suggest that non-contrast protocols appear preferable in most clinical settings.

Diagnostic accuracy data were sparse, with only 2 studies reporting quantitative diagnostic performance metrics.^3^^,^^15^ Both demonstrated encouraging sensitivity and specificity for detecting bone and extrapulmonary metastases, comparable to established reference standards such as CT and PET-CT. Karaarslan et al^3^ reported excellent sensitivity (100%) and specificity (96.3%) for extrapulmonary metastases in osteosarcoma, while Aryal et al^15^ showed high accuracy for bone metastases in both osteosarcoma and Ewing sarcoma. These findings support the potential of WB-MRI as a reliable modality for comprehensive staging. However, the paucity of diagnostic accuracy studies remains a major limitation. Most available data are descriptive and lack systematic validation against histopathology or standardized reference imaging.

One of the main concerns regarding the use of WB-MRI for cancer staging is the detection of lung metastases, with scarce evidence supporting the use of this imaging modality. WB-MRI has shown 80% sensitivity, 97.73% specificity, and 95.92% accuracy for detecting lung metastases from breast cancer.^25^ Unfortunately, pulmonary metastases represent the most frequent site of distant spread in sarcoma and identification and management of lung metastases can influence short-term survival in both bone and soft tissue sarcoma.^26^ Further, the diagnostic performance of this exam in detecting metastases would potentially require longer scan times with dedicated loco-regional protocols in addition to the whole-body study, making the examination less suitable. Currently, CT remains the optimal imaging modality and surveillance schedule for pulmonary monitoring,^27^ with some investigations having suggested improved survival outcomes with CT-based follow-up,^28^ while a randomized controlled trial demonstrated no survival advantage compared with radiographic surveillance,^29^ but with increased cost and radiation exposure.^30^^,^^31^ Current international guidelines remain inconsistent regarding both imaging modality and follow-up intervals. For example, the National Comprehensive Cancer Network favours CT surveillance in bone^32^ and soft tissue sarcoma,^33^ while acknowledging that outcome benefits have not been demonstrated. In contrast, the European Society for Medical Oncology and the Musculoskeletal Tumor Society endorse broader ranges of acceptable strategies.^34^

Our review highlights some practical advantages of WB-MRI in musculoskeletal oncology. First, it avoids ionizing radiation, a particularly important consideration in paediatric and adolescent populations requiring serial imaging. However, in this population, prolonged scan times and the potential need for sedation represent practical challenges; therefore, optimized, time-efficient WB-MRI protocols and motion-robust sequences are essential to improve feasibility. Second, it provides a single comprehensive examination capable of assessing both loco-regional and distant disease, potentially reducing the need for multiple imaging modalities. Third, WB-MRI offers superior soft tissue contrast compared to CT and bone scintigraphy, it is considered superior to PET/CT and CT for bone locations of disease in melanoma, multiple myeloma, and lobular breast carcinoma, and is the gold standard for cancers in pregnancy and in patients with a genetic predisposition to cancer.^35^ Nevertheless, several barriers hinder routine implementation. These include restricted availability of WB-MRI expertise, limited scanner time in high-volume centres and the lack of standardized protocols. Standardization of acquisition parameters and reporting frameworks should draw inspiration from existing multicentre initiatives such as MY-RADS,^36^ MET-RADS-P,^37^ and ONCO-RADS,^38^ which have demonstrated the feasibility of harmonized WB-MRI protocols and structured data reporting across different oncologic applications. Additionally, patient tolerance, particularly in younger children and those with advanced disease, remains a practical concern. Sedation may be required in select paediatric cases, further complicating logistics.

When considering the studies according to their clinical application, distinct scenarios can be identified. For initial staging, WB-MRI was mainly used to assess the extent of disease and detect distant metastases, demonstrating high sensitivity for bone and extrapulmonary lesions in comparison with conventional imaging. Regarding treatment response, only a minority of studies included post-therapy evaluations, reporting the potential of WB-MRI to differentiate viable tumour tissue from post-treatment changes through STIR and diffusion-weighted sequences. In the follow-up/surveillance setting, WB-MRI proved useful for detecting extrapulmonary recurrences and osseous metastases, although its systematic use is still limited to specialized centres.

Across the 5 studies focusing on myxoid liposarcoma, WB-MRI consistently demonstrated high sensitivity for detecting extra-pulmonary and particularly osseous metastases, both at diagnosis and during surveillance. Four of 5 studies primarily evaluated WB-MRI in the *initial staging* setting,^10^^,^^16^^,^^18^^,^^20^ 2 studies also incorporated *post-treatment follow-up*,^10^^,^^20^ 1 included *restaging after relapse,*^16^ and 1 focused chiefly on *annual surveillance.*^17^ Reported metastatic rates at diagnosis ranged from 25% to 27%,^16^^,^^18^ with lesions predominantly extrapulmonary, asymptomatic, and frequently occult on CT.^10^^,^^16–18^ WB-MRI identified additional bone and soft-tissue metastases not visualized on contemporaneous CT in up to 78% of cases,^10^^,^^16^^,^^18^ and this improved detection directly altered management in staging settings, particularly when occult metastatic disease was discovered prior to definitive local treatment.^16^^,^^18^ In surveillance cohorts, WB-MRI facilitated early diagnosis of extrapulmonary metastases—often months to years before pulmonary involvement or symptom onset.^10^^,^^17^ Additionally, sequence-optimization work demonstrated that STIR-only WB-MRI significantly reduces acquisition time while maintaining excellent sensitivity for osseous metastases.^20^ Overall, the available data suggest that WB-MRI may offer greater sensitivity than CT for detecting extrapulmonary and osseous metastases in myxoid liposarcoma, both at initial staging and during follow-up, although the evidence remains limited and further studies are needed to confirm these findings.

The available studies showed substantial heterogeneity in both clinical and technical aspects, including tumour histology, patient age range and MRI field strength (1.5T vs 3T). These factors likely contributed to variability in diagnostic performance, since different sarcoma subtypes and scanner configurations may affect lesion conspicuity, signal-to-noise ratio and the presence of artefacts. Due to such heterogeneity and the limited number of studies reporting complete diagnostic accuracy data (*n = *2), a quantitative meta-analysis could not be performed. In this regard, the limitations of the included studies must be emphasized. Most were retrospective and single-center, with heterogeneous protocols and small sample sizes. Reference standards were inconsistently applied, ranging from CT and PET-CT to clinical follow-up, which may have introduced verification bias. Furthermore, the predominance of studies on myxoid liposarcoma may limit generalizability to other sarcoma subtypes. Finally, only a minority of studies reported diagnostic performance metrics, precluding pooled analysis or meta-analysis.

Future research should prioritize the prospective validation of standardized WB-MRI protocols across multiple centres and tumour subtypes. Comparative studies directly assessing WB-MRI against PET-CT, including hybrid PET-MRI systems, are warranted to clarify their respective roles in staging, treatment response assessment, and long-term surveillance. Additionally, artificial intelligence-based post-processing tools hold promise for automating lesion detection, segmentation and quantitative evaluation, potentially reducing reader variability and reporting time.^39^^,^^40^ Cost-effectiveness analyses are also needed to determine whether WB-MRI can reduce reliance on multiple radiation-based imaging modalities in routine clinical pathways.

In conclusion, WB-MRI represents a promising technique for the initial staging and follow-up of primary musculoskeletal tumours, offering comprehensive, radiation-free assessment with high sensitivity for metastatic disease. Our findings indicate that unenhanced protocols remain the most practical approach, while the role of contrast is limited. Differences in imaging protocols across studies likely influenced diagnostic performance. Most protocols relied on unenhanced T1- and STIR-weighted sequences, which provide robust detection of bone marrow and soft tissue lesions, but may have limited sensitivity for small or subtle metastases compared with contrast-enhanced or diffusion-weighted acquisitions. Conversely, the inclusion of multiple contrast-enhanced or DWI sequences can improve lesion conspicuity, but often at the cost of longer scan times, increased motion artefacts and reduced patient compliance, particularly in paediatric or frail populations. Such variability in sequence selection and acquisition parameters inevitably affects comparability across studies and contributes to the heterogeneity of reported diagnostic accuracy. Examination duration remains a major challenge, with scan times varying considerably across studies. Although early diagnostic performance data are encouraging, the overall evidence base is restricted to small, heterogeneous studies. Standardization of protocols and prospective validation in larger, multicentre cohorts are essential to fully establish the role of WB-MRI in musculoskeletal oncology. At present, WB-MRI cannot be recommended as a routine alternative to established imaging modalities such as CT, PET-CT, or bone scintigraphy. Nonetheless, its potential benefits warrant further investigation.

## Supplementary Material

tqag009_Supplementary_Data

## References

[1] Cruz IAN , Fayad LM, Ahlawat S, et al Whole-Body MRI in musculoskeletal oncology: a comprehensive review with recommendations. Radiol Imaging Cancer. 2023; 5:e220107. 37144975 10.1148/rycan.220107 PMC10240252

[2] Stanborough R , Demertzis JL, Wessell DE, et al ACR appropriateness criteria^®^ malignant or aggressive primary musculoskeletal tumor-staging and surveillance: 2022 update. J Am Coll Radiol. 2022;19:S374-S389. 36436964 10.1016/j.jacr.2022.09.015

[3] Karaarslan E , Alis D, Basar Y, et al The role of whole-body magnetic resonance imaging in assessing extrapulmonary metastases in osteosarcoma staging and restaging: a pilot study. J Comput Assist Tomogr. 2023;47:629-636. 36944103 10.1097/RCT.0000000000001455

[4] Albano D , Stecco A, Micci G, et al Whole-body magnetic resonance imaging (WB-MRI) in oncology: an Italian survey. Radiol Med. 2021; 126:299-305. 32572763 10.1007/s11547-020-01242-7

[5] Albano D , Patti C, La Grutta L, et al Osteonecrosis detected by whole body magnetic resonance in patients with Hodgkin lymphoma treated by BEACOPP. Eur Radiol. 2017;27:2129-2136. 27519911 10.1007/s00330-016-4535-8

[6] Messiou C , Porta N, Koh D-M, et al Whole body MRI by MY-RADS for imaging response assessment in multiple myeloma. Blood Cancer J. 2025;15:122. 40675973 10.1038/s41408-025-01327-4 PMC12271311

[7] Parillo M , Mallio CA. The whole-body MRI reporting and data system guidelines for prostate cancer (MET-RADS-P), multiple myeloma (MY-RADS), and cancer screening (ONCO-RADS). Cancers (Basel). 2025;17:275. 39858056 10.3390/cancers17020275 PMC11763526

[8] Greer MLC , States LJ, Malkin D, Voss SD, Doria AS. Update on whole-body MRI surveillance for pediatric cancer predisposition syndromes. Clin Cancer Res. 2024;30:5021-5033. 39287924 10.1158/1078-0432.CCR-24-1374

[9] Que FVF , Ishak NDB, Li S-T, et al Utility of whole-body magnetic resonance imaging surveillance in children and adults with cancer predisposition syndromes: a retrospective study. JCO Precis Oncol. 2025;9:e2400642. 40138602 10.1200/PO-24-00642 PMC11949220

[10] Gorelik N , Reddy SMV, Turcotte RE, et al Early detection of metastases using whole-body MRI for initial staging and routine follow-up of myxoid liposarcoma. Skeletal Radiol. 2018;47:369-379. 29275455 10.1007/s00256-017-2845-9

[11] Kalus S , Saifuddin A. Whole-body MRI vs bone scintigraphy in the staging of ewing sarcoma of bone: a 12-year single-institution review. Eur Radiol. 2019;29:5700-5708. 30915559 10.1007/s00330-019-06132-9

[12] Liberati A , Altman DG, Tetzlaff J, et al The PRISMA statement for reporting systematic reviews and meta-analyses of studies that evaluate health care interventions: explanation and elaboration. Ann Intern Med. 2009;151:W65-W94. 19622512 10.7326/0003-4819-151-4-200908180-00136

[13] Kmet LM , Lee RC, Cook LS. Standard Quality Assessment Criteria for Evaluating Primary Research Papers from a Variety of Fields. HTA Initiative #13. 2004. Alberta Heritage Foundation for Medical Research (AHFMR). https://www.ihe.ca/files/standard_quality_assessment_criteria_for_evaluating_primary_research_papers_from_a_variety_of_fields.pdf.

[14] Van der Woude HJ , Flipsen M, Welsink C, Van der Zwan AL, Ham SJ. Is total-body MRI useful as a screening tool to rule out malignant progression in patients with multiple osteochondromas? Results in a single-center cohort of 319 adult patients. Skeletal Radiol. 2024;53:141-150. 37338590 10.1007/s00256-023-04389-2

[15] Aryal A , Kumar VS, Shamim SA, Gamanagatti S, Khan SA. What is the comparative ability of 18F-FDG PET/CT, 99mTc-MDP skeletal scintigraphy, and whole-body MRI as a staging investigation to detect skeletal metastases in patients with osteosarcoma and ewing sarcoma? Clin Orthop Relat Res. 2021;479:1768-1779. 33635285 10.1097/CORR.0000000000001681 PMC8277296

[16] Stevenson JD , Watson JJ, Cool P, et al Whole-body magnetic resonance imaging in myxoid liposarcoma: a useful adjunct for the detection of extra-pulmonary metastatic disease. Eur J Surg Oncol. 2016;42:574-580. 26831007 10.1016/j.ejso.2015.12.011

[17] Gouin F , Renault A, Bertrand-Vasseur A, et al Early detection of multiple bone and extra-skeletal metastases by body magnetic resonance imaging (BMRI) after treatment of myxoid/round-cell liposarcoma (MRCLS). Eur J Surg Oncol. 2019;45:2431-2436. 31447287 10.1016/j.ejso.2019.08.014

[18] Dewaguet J , Beaujot J, Leguillette C, et al Apport de l’IRM corps entier au bilan initial du liposarcome myxoïde. Bull Cancer. 2023;110:1015-1026. 37507239 10.1016/j.bulcan.2023.05.009

[19] Jurik AG , Jørgensen PH, Mortensen MM. Whole-body MRI in assessing malignant transformation in multiple hereditary exostoses and enchondromatosis: audit results and literature review. Skeletal Radiol. 2020;49:115-124. 31273432 10.1007/s00256-019-03268-z

[20] Chien A , Zuppan CW, Lei L, et al Short tau inversion recovery magnetic resonance imaging for staging and screening in myxoid liposarcoma. J Orthop. 2019;16:206-210. 30906124 10.1016/j.jor.2019.02.023 PMC6411606

[21] Albano D , Patti C, Lagalla R, Midiri M, Galia M. Whole‐body MRI, FDG‐PET/CT, and bone marrow biopsy, for the assessment of bone marrow involvement in patients with newly diagnosed lymphoma. J Magn Reson Imaging. 2017;45:1082-1089. 27603267 10.1002/jmri.25439

[22] Albano D , La Grutta L, Grassedonio E, et al Pitfalls in whole body MRI with diffusion weighted imaging performed on patients with lymphoma: what radiologists should know. Magn Reson Imaging. 2016;34:922-931. 27114337 10.1016/j.mri.2016.04.023

[23] Messina C , Bignone R, Bruno A, et al Diffusion-weighted imaging in oncology: an update. Cancers (Basel). 2020;12:1493. 32521645 10.3390/cancers12061493 PMC7352852

[24] Pozzi G , Albano D, Messina C, et al Solid bone tumors of the spine: diagnostic performance of apparent diffusion coefficient measured using diffusion‐weighted MRI using histology as a reference standard. J Magn Reson Imaging. 2018;47:1034-1042. 28755383 10.1002/jmri.25826

[25] Hottat NA , Badr DA, Ben Ghanem M, et al Assessment of whole-body MRI including diffusion-weighted sequences in the initial staging of breast cancer patients at high risk of metastases in comparison with PET-CT: a prospective cohort study. Eur Radiol. 2024;34:165-178. 37555959 10.1007/s00330-023-10060-0

[26] Predina JD , Puc MM, Bergey MR, et al Improved survival after pulmonary metastasectomy for soft tissue sarcoma. J Thorac Oncol. 2011;6:913-919. 21750417 10.1097/JTO.0b013e3182106f5c

[27] Tepper SC , Holten AK, Jeffe DB, et al Examining patient perspectives on sarcoma surveillance: the sarcoma surveillance survey. Surg Oncol. 2022;45:101861. 36270157 10.1016/j.suronc.2022.101861 PMC9729379

[28] Cho HS , Park IH, Jeong WJ, Han I, Kim HS. Prognostic value of computed tomography for monitoring pulmonary metastases in soft tissue sarcoma patients after surgical management: a retrospective cohort study. Ann Surg Oncol. 2011;18:3392-3398. 21537873 10.1245/s10434-011-1705-4

[29] Puri A , Gulia A, Hawaldar R, Ranganathan P, Badwe RA. Does intensity of surveillance affect survival after surgery for sarcomas? Results of a randomized noninferiority trial. Clin Orthop Relat Res. 2014;472:1568-1575. 24249538 10.1007/s11999-013-3385-9 PMC3971232

[30] Croswell JM , Baker SG, Marcus PM, Clapp JD, Kramer BS. Cumulative incidence of false-positive test results in lung cancer screening. Ann Intern Med. 2010;152:505-512. 20404381 10.7326/0003-4819-152-8-201004200-00007

[31] Berrington de González A , Mahesh M, Kim K-P, et al Projected cancer risks from computed tomographic scans performed in the United States in 2007. Arch Intern Med. 2009;169:2071-2077. 20008689 10.1001/archinternmed.2009.440 PMC6276814

[32] Biermann JS , Chow W, Reed DR, et al NCCN guidelines insights: bone cancer, version 2.2017. J Natl Compr Canc Netw. 2017;15:155-167. 28188186 10.6004/jnccn.2017.0017

[33] von Mehren M , Randall RL, Benjamin RS, et al Soft tissue sarcoma, version 2.2018, NCCN clinical practice guidelines in oncology. J Natl Compr Canc Netw. 2018;16:536-563. 29752328 10.6004/jnccn.2018.0025

[34] Gronchi A , Miah AB, Dei Tos AP, et al; ESMO Guidelines Committee, EURACAN and GENTURIS. Soft tissue and visceral sarcomas: ESMO-EURACAN-GENTURIS clinical practice guidelines for diagnosis, treatment and follow-up. Ann Oncol. 2021;32:1348-1365. 34303806 10.1016/j.annonc.2021.07.006

[35] Lecouvet FE , Chabot C, Taihi L, Kirchgesner T, Triqueneaux P, Malghem J. Present and future of whole-body MRI in metastatic disease and myeloma: how and why you will do it. Skeletal Radiol. 2024;53:1815-1831. 39007948 10.1007/s00256-024-04723-2 PMC11303436

[36] Rata M , Blackledge M, Scurr E, et al Implementation of whole-body MRI (MY-RADS) within the OPTIMUM/MUKnine multi-centre clinical trial for patients with myeloma. Insights Imaging. 2022;13:123. 35900614 10.1186/s13244-022-01253-0 PMC9334517

[37] Padhani AR , Lecouvet FE, Tunariu N, et al METastasis reporting and data system for prostate cancer: practical guidelines for acquisition, interpretation, and reporting of whole-body magnetic resonance imaging-based evaluations of multiorgan involvement in advanced prostate cancer. Eur Urol. 2017;71:81-92. 27317091 10.1016/j.eururo.2016.05.033 PMC5176005

[38] Petralia G , Koh D-M, Attariwala R, et al Oncologically relevant findings reporting and data system (ONCO-RADS): guidelines for the acquisition, interpretation, and reporting of whole-body MRI for cancer screening. Radiology. 2021;299:494-507. 33904776 10.1148/radiol.2021201740

[39] Gitto S , Serpi F, Albano D, et al AI applications in musculoskeletal imaging: a narrative review. Eur Radiol Exp. 2024;8:22. 38355767 10.1186/s41747-024-00422-8 PMC10866817

[40] Zanardo M , Visser JJ, Colarieti A, et al; European Society of Radiology (ESR). Impact of AI on radiology: a EuroAIM/EuSoMII 2024 survey among members of the European society of radiology. Insights Imaging. 2024;15:240. 39373853 10.1186/s13244-024-01801-w PMC11458846

