# Supplementary material for: Whole-body MRI for staging and follow-up of primary musculoskeletal tumours: a systematic review
Source: Br J Radiol. 2026 Jan 8;99(1180):747–54. doi: 10.1093/bjr/tqag009 (PMC13070648; doi:10.1093/bjr/tqag009)
Supplement: tqag009_Supplementary_Data [file tqag009_supplementary_data.zip › Supplementary Material.docx]

**Supplementary Material – Search Strategy and Study Selection**

*Embase search string*

(('sarcoma'/exp OR 'histioblastoma' OR 'histiosarcoma' OR 'malignant sarcoma' OR 'mast cell sarcoma' OR 'mast-cell sarcoma' OR 'neoplasm, mesenchymal' OR 'sarcoma' OR 'sarcoma e 100' OR 'sarcoma l 1' OR 'sarcoma sa 1' OR 'sarcoma tawa' OR 'sarcoma, mast-cell') OR ('osteosarcoma'/exp OR 'osteo-chondrosarcoma' OR 'osteo-sarcoma' OR 'osteogenic sarcoma' OR 'osteoid sarcoma' OR 'osteolytic sarcoma' OR 'osteosarcoma' OR 'primary osteogenic sarcoma' OR 'sarcoma, osteogenic') OR ('Ewing sarcoma'/exp OR 'Ewing neoplasm' OR 'Ewing sarcoma' OR 'Ewing tumor' OR 'Ewing tumour' OR 'Ewing`s neoplasm' OR 'Ewing`s sarcoma' OR 'Ewing`s tumor' OR 'Ewing`s tumour' OR 'Ewings sarcoma' OR 'Ewings tumor' OR 'Ewings tumour' OR 'bone sarcoma, ewing' OR 'sarcoma, Ewing' OR 'sarcoma, Ewing`s' OR 'skeletal Ewing sarcoma') OR 'chondrosarcoma'/exp OR ('soft tissue sarcoma'/exp OR 'sarcoma, soft tissue' OR 'soft part sarcoma' OR 'soft tissue sarcoma') OR ('musculoskeletal tumor'/exp OR 'locomotor system tumor' OR 'locomotor system tumour' OR 'musculo-skeletal tumor' OR 'musculo-skeletal tumour' OR 'musculoskeletal neoplasia' OR 'musculoskeletal neoplasm' OR 'musculoskeletal system neoplasia' OR 'musculoskeletal system tumor' OR 'musculoskeletal tumor' OR 'musculoskeletal tumour' OR 'neoplasms of musculoskeletal' OR 'tumors of musculoskeletal') OR 'primary bone tumour') AND (('whole body MRI'/exp OR 'whole body MRI' OR 'whole body magnetic resonance' OR 'whole body magnetic resonance imaging') OR 'whole-body MRI' OR 'WB-MRI')

*Pubmed*

(((((((((("sarcoma"[Title/Abstract]) OR ("malignant sarcoma"[Title/Abstract])) OR ("osteosarcoma"[Title/Abstract])) OR ("primary osteogenic sarcoma"[Title/Abstract]))) OR ("ewing sarcoma"[Title/Abstract])) OR ("bone sarcoma"[Title/Abstract])) OR ("chondrosarcoma"[Title/Abstract])) OR ("soft tissue sarcoma"[Title/Abstract] OR "soft tissue sarcoma accounting"[Title/Abstract])) OR ("musculoskeletal tumor"[Title/Abstract] OR "musculoskeletal tumor detection"[Title/Abstract])) AND ("whole body mri"[Title/Abstract] OR "whole body mri data"[Title/Abstract] OR "whole body mri dwi"[Title/Abstract] OR "whole body mri images"[Title/Abstract] OR "whole body mri protocol"[Title/Abstract] OR "whole body mri scanner"[Title/Abstract] OR "whole body mri screening"[Title/Abstract] OR "whole body mri systems"[Title/Abstract] OR "whole body mri techniques"[Title/Abstract] OR "whole body musculoskeletal model"[Title/Abstract])

*Study selection*

- All records were imported into a reference manager and duplicates were removed.
- Titles and abstracts were independently screened by two reviewers to identify potentially relevant studies.
- Full texts of selected articles were then assessed for eligibility according to predefined inclusion and exclusion criteria.
- Discrepancies were resolved through discussion or by consulting a third reviewer.
- Reference lists of included studies and relevant reviews were hand-searched to identify additional eligible articles.
